# Body fat, eating behaviours, and well-being as predictors of negative body talk among college students: a moderation analysis by sex

**DOI:** 10.1186/s12889-026-27558-z

**Published:** 2026-04-28

**Authors:** Guifang Su, Zhimin Yi, Ying Wang, Chunmei Wu, Ming Hao, Jiayi Luo, Yunquan Cai

**Affiliations:** School of Public Health and Health Management, Gannan Medical University, University Park, Rongjiang new area, Ganzhou City, Jiangxi Province 341000 China

**Keywords:** Body fat, Negative body talk, Eating behaviour, Well-being

## Abstract

**Background:**

Negative body talk (e.g., body shaming) among college students exacerbates mental health risks (e.g., eating disorders, depression) and peer body anxiety through social norms. This study examined multifactorial mechanisms and gender differences with an aim informing mental health interventions.

**Methods:**

This study was conducted on 789 college students (260 males and 529 females), 18–23 years, from a university in Ganzhou City, Jiangxi Province. Data were collected by standardised instruments: body fat percentage, muscle mass, and height were measured using a body composition meter (Tanita BC-610) and a height scale; physical activity level was quantified using the Physical Activity Rating Scale (PARS-3); and dietary behaviours were measured using the Three Factor Eating Questionnaire Revised (TFEQ-R21v2). The scale contains three dimensions: Cognitive Restraint, Emotional Eating, and Uncontrolled Eating. Subjective well-being was measured using the WHO-5 Happiness Index.

**Results:**

Sex differences existed in BMI, body fat, muscle mass, and physical activity ( *P* < 0.05). Body fat (β = 0.26) and restrictive diet (β = 0.51) positively predicted negative body talk ( *P* < 0.001), while happiness (β=−0.09) and uncontrolled eating (β=−0.08) showed negative associations ( *P* < 0.05). Men exhibited stronger effects of body fat (β = 0.16) and restrictive diet (β = 0.49) ( *P* < 0.01). In women, body fat (β = 0.36), restrictive diet (β = 0.50), and emotional eating (β = 0.10) correlated more strongly with lower well-being ( *P* < 0.01). High body fat-high restriction, low body fat-high well-being, and moderate groups differed significantly in BMI, body fat, eating behaviors, and well-being ( *P* < 0.001).

**Conclusion:**

Chinese university students show a high prevalence of negative body talk, with females significantly higher than males. Body fat and restrictive eating positively predict negative discourse, while happiness and non-restrictive eating negatively predict it. The high body fat-high restrictive eating group, scoring highest on negative discourse and lowest on happiness, is a priority for psychological intervention.

## Introduction

Recently, the phenomenon of negative body talk (NOT) has gained global prominence and has become a significant concern threatening public mental health [1]. Such discourse employs derogatory language to pass negative judgement on one’s own or others’ physical appearance (e.g., ‘My figure is dreadful,’ ‘I’m unworthy of love unless I reach my target weight’), thereby intensifying body dissatisfaction and anxiety. This perpetuates a vicious cycle of societal pressure, triggering collective anxiety disorders and ultimately precipitating a mass psychological crisis. International studies have shown that approximately 76% of European and American adolescent females engage in negative body talk discussions because of body size anxiety, with 43% indicating that such discussions lead directly to binge eating or excessive dieting behaviours [2]. In East Asian societies influenced by collectivist culture, negative shelf-talk is often presented in the form of ‘self-deprecation’ (for example referring to oneself as a ‘happy chubby boy’). This is both an attempt to gain acceptance through humour and exacerbates psychological conflict by continually reinforcing the negative self-perception [3]. This seemingly humorous interaction reinforces the occurrence of weight-related stigma.

With the rapid economic development and lifestyle changes in China, the population has also shown a continuously rising trend toward higher body weight. Weight-related concerns among Chinese college students have become a growing social concern, with regional surveys showing that the proportion of youth in higher BMI categories has exceeded 20% [4]. More alarmingly, negative discussions about body size has given rise to a particular subculture, with approximately 65% of college students admitting to experiencing body size anxiety often expressed through self-deprecation [5]. For instance, a student might joke, ‘I have to turn sideways to get through the doorway,’ or refer to themselves after a meal as ‘a waste of society’s resources’—statements that implicitly equate body size with personal worth. Such humour treats body size as a legitimate target for ridicule, thereby normalising underlying biases and internalising societal discrimination as an irrefutable personal flaw. Questioning this humour risks being dismissed as lacking wit. This seemingly humorous interaction reinforces the occurrence of weight-related stigma.

Social media platforms compound the problem. On Chinese version of Weibo and Chinese version of Rednote—China’s two most influential social media platforms—topics such as ‘weight loss challenges’ and ‘body comparison’ where users share lifestyle content and engage in appearance-related discussions have garnered billions of views, with the overwhelming majority of readers being female [6]. Moreover, much of this content involves harsh self-scrutiny of body image, exposing users to frequent negative body image messages—defined as content that criticises, compares, or expresses dissatisfaction with one’s appearance, often reinforcing unrealistic aesthetic standards [6]. This phenomenon, often termed “fat talk” in academic literature, refers to ritualistic conversations about one’s own or others’ bodies that perpetuate negative self-perceptions [6]. This results in users being frequently exposed to negative body image messages, with significantly higher frequencies of negative body talk (i.e., verbal expressions of dissatisfaction or self-criticism about one’s body, also termed ‘fat talk’) compared to the general population [5]. Notably, the ‘social transmission’ of negative body talk has been experimentally demonstrated: a follow-up study showed that individuals exposed to group body degradation discussions experienced a 19% increase in body dissatisfaction over 24 hours and a higher likelihood of developing collective disordered eating behaviors within a small female group [7] .

Students are at a particular risk of weight gain to factors such as uncontrolled eating in the early years of independent living, academic pressures, and the portrayal of “ideal body types” on social media [8]. However, the impact of weight-related concerns on university students extends far beyond the physical realm. With the emergence of “negative body talk” (NOT), people have begun using derogatory language to discuss weight gain (such as “my belly resembles a swimming ring” or “I must slim down to 90 g to count as having successfully lost weight.”). This negative body talk, which expresses excessive anxiety about one’s own or others’ body image through disparaging language, is becoming an invisible killer threatening mental health [1]. Such remarks are rooted in society’s cultural glorification of the ‘thin ideal’ [9] and perpetuate a vicious cycle through self-objectification: individuals constantly monitor whether their bodies conform to societal norms, generating anxiety and shame, while simultaneously releasing pressure through negative comments [9]. Individuals who frequently engage in negative talk have a 2.3-fold higher risk of eating disorders than the general population [10] and may even indirectly increase the risk of cardiovascular disease by exacerbating chronic inflammation through a neuroendocrine stress response [11]. Moreover, significant gender differences emerge in this phenomenon: women, exposed to social media hashtags such as ‘A4 waist’ and ‘manga legs,’ are more inclined toward self-deprecation and frequently engage in ‘weight loss challenges’ as a form of bodily self-discipline—a practice particularly prevalent among contemporary female university students in China [12]. Empirical studies have shown that about 80% of the respondents have participated in such activities, with 77% being female. More than half of them have experienced disorders in their self-evaluation system [13], while men focus on the implicit anxiety of ‘not having enough muscle mass’ [14]. University students face excessive social media usage, peer pressure and academic stress, factors which render negative discourse a social tool for alleviating anxiety [15]. More seriously, such talk appears to be socially contagious, with evidence showing a 19% increase in body dissatisfaction within 24 hours among individuals exposed to derogatory group discussions [2]. This may evolve into a collective psychosocial crisis within small groups of women.

Research has shown that both emotional and restrictive eating are significantly associated with negative body talk. For example, restrictive eating behaviours may stem from excessive weight concerns, which themselves may exacerbate an individual’s dissatisfaction with body image, which in turn may lead to more negative body talk [16]. Additionally, emotional eating (eating in response to emotional states such as stress or anxiety) and uncontrolled eating (the tendency to overeat in response to external food cues or when experiencing a loss of control) may temporarily alleviate negative emotions. Nonetheless, these unhealthy eating behaviours may also negatively impact body weight and psychological well-being [17]. Women are more likely to engage in unhealthy eating behaviours due to emotional fluctuations, which in turn affect their weight and mental health [8].

Happiness, as a key psychological indicator measuring an individual’s life satisfaction and positive emotional experiences, is associated with body weight status and dietary behaviours [14]. This helps explain why individuals with higher levels of well-being typically exhibit greater life satisfaction and positive emotions, thereby enabling them to cope more effectively with life’s pressures and challenges. Research suggests that higher well-being may enhance an individual’s psychological resilience, enabling them to cope better with weight-related social pressures, thereby reducing the incidence of negative body talk [18]. Conversely, lower well-being may make individuals more susceptible to negative external evaluations, which, in turn, exacerbates the propensity for negative body talk [19]. The protective effect of well-being on mental health has not been fully validated in the context of weight-related stress.

College students are at a critical stage of shaping identity and values, and concerns about body size and weight are of significant importance. They face multiple pressures, such as academic competition, the rendering of the ‘ideal body’ on social media, and body comparisons among peers. This makes higher body weight not only a health concern but also a factor profoundly affecting their psychosocial adaptations [8]. However, most existing studies have focused on the physiological health effects of higher body weight, and there is still a significant gap in exploring its psychosocial mechanisms, especially when it comes to differences between sexes [20]. The present study aimed to explore the relationship between body fat, eating behaviours, well-being, and negative body talk among college students, and to examine how these factors collectively influence students’ perceptions and discussions of body weight. The findings of this study will provide empirical evidence for mental health education in higher education institutions, facilitating its transition from a focus on “weight control” towards “social cognitive restructuring”. Furthermore, the conclusions hold significant reference value for promoting the physical and mental wellbeing of university students and fostering a positive campus environment.

## Research methods

### Research participants

This study was conducted at a comprehensive university in Jiangxi Province.A total of 800 participants were recruited via announcements displayed in student dormitories and study areas. An online questionnaire was created using Chinese version of Questionnaire Star, and a QR code was generated and distributed to the participants. During students’ evening study sessions in the classroom, Professional measurement technicians will collect participants’ physical measurements in a classroom setting and guide them through the online questionnaire. Students under the age of 18 are excluded due to their small numbers. Finally, 789 students (260 men and 529 women) aged 18–23 years, completed the survey, and their data were included in the analysis.

### Measurement

Height was measured with an accuracy of 0.1 cm using a portable rangefinder (Seca 213; Germany). Body weight (0.1 kg accuracy), fat percentage, and muscle mass (0.1 kg accuracy) were measured using a body composition meter (Tanita BC-610, Japan). Body Mass Index (BMI) (Kg/m^2^) was calculated using height and weigh, and BMI (Body Mass Index) is usually classified according to the following criteria: wasting: BMI < 18.5; normal: 18.6 ≤ BMI ≤ 24.9; overweight: 25.0 ≤ BMI < 29.9; and obese: BMI ≥ 30.0.

#### Physical activity

Physical activity was measured using the Physical Activity Rating Scale-3 (PARS-3), a five-item self-report scale covering duration, intensity, and frequency [18]. Ratings were given on a scale from 1 to 5. The total physical activity score (i.e., amount of exercise) was calculated using the following formula: intensity × (duration − 1) × frequency. The total physical activity score ranged from 0 to 100. Based on this total score, physical activity was categorized into four levels: no exercise (score ≤ 4), low exercise (score 5–19), moderate exercise (score 20–42), and high exercise (score ≥ 43).The scale demonstrated good internal consistency, with a Cronbach’s alpha of 0.83.

#### Eating behaviour

The TFEQ-R21v2 (Twenty-Item Food Intake and Eating Behavior Questionnaire-Revised 21 version 2) scale was used to assess students’ eating behaviours. The TFEQ-R21v2 is a widely used tool in eating behaviour research and has been validated for good reliability and validity [21]. Cognitive Restraint: a behaviour in which an individual actively controls calorie intake through cognitive strategies (e.g., food choice, meal planning). Emotional Eating: assesses eating behaviours in response to emotional states such as stress, anxiety or boredom. Uncontrolled Eating: assesses an individual’s tendency to respond automatically to external food cues in the absence of hunger. Each dimension consists of 7 items rated on a 5-point Likert scale (from 1= ‘never’ to 5 =‘always’). The total score ranges from 21 to105, with higher scores indicating more severe eating behaviour problems. The scale demonstrated good internal consistency, with a Cronbach’s alpha of 0.85.

#### Happiness

The WHO-5, formally known as the World Health Organisation Five-Item Happiness Index, was developed by Bech et al.(2003) [22], and is commonly used to measure subjective well-being. The WHO-5 questionnaire comprises five items, each scored on a six-point scale ranging from 0 (never) to 6 (all the time), to assess the relevant items over the preceding two weeks. By adding items to obtain a total score, a higher score indicates better mental health. The Cronbach’s α coefficient for well-being was 0.88, indicating excellent internal consistency (> 0.8) [19]. Numerous studies have demonstrated a significant positive correlation between body image satisfaction and WHO-5 scores, providing empirical support for incorporating the WHO-5 into models assessing the interplay between body fat, body image, and well-being [23].

#### Body Talk Scale

We employed the Body Talk Scale (BTS), designed for Asian populations, to measure participants’ perceptions of two types of body dissatisfaction by assessing their cross-gender negative body talk and positive body talk [24]. The scale comprises three dimensions: negative body talk, negative muscularity talk, and positive body talk. Negative body talk comprises five items: (1) I need to lose weight; (2) I feel fat; (3) My clothes are too tight because of my weight; (4) I should stop eating foods that make me fat; (5) I need to exercise more to lose weight. Scored on a six-point Likert scale (1 = never; 6 = always), higher total scores indicate greater body dissatisfaction. All items were scored positively. In this study, the Cronbach’s alpha coefficient for this scale was 0.79, indicating acceptable internal consistency.

### Statistical analyses

Independent t-tests were conducted to examine sex differences in participants’ BMI, body fat, muscle mass, physical activity, and dietary scores. In multiple regression analysis, negative body talk were employed as the dependent variable. Body fat, emotional eating, uncontrolled eating, restricted eating, physical activity scores, and total well-being scores were included as predictor variables. A p-value of less than 0.05 was considered statistically significant.

To further explore differential patterns in negative body talk among university students with distinct characteristics, latent class analysis (LCA) was conducted using Mplus 8.10 software [25]. LCA is an individual-centred statistical method that classifies individuals into mutually exclusive latent categories based on their response patterns across multiple observed indicators. The LCA analysis in this study incorporated the following classification indicators: body fat percentage, restrictive eating score, emotional eating score, uncontrolled eating score, overall well-being score, and physical activity score. Following classification, the Bolck-Croon-Hagenaars (BCH) method [26] was employed to compare differences in negative body talk scores across distinct latent categories, thereby revealing which combinations of characteristics predispose groups to greater engagement in negative body talk.

## Results

Men’s BMI was significantly higher than women’s (*P* < 0.001; Table 1) while physical activity levels were significantly lower in men than women (*P* < 0.001).

**Table 1:** Participants’ characteristics

Characteristics	Mean ± SD or *n* (%)		*P*
	Men (*n* = 260)	Women (*n* = 529)	
BMI	22.7 ± 4.3	21.2 ± 3.5	< 0.001
Body fat	16.5 ± 8.2	26.1 ± 6.7	< 0.001
Muscle mass	48.0 ± 11.9	35.8 ± 5.8	< 0.001
BMI category			
Wasting	45(17.3)	119 (22.5)	< 0.001
Normal	152 (58.5)	343 (64.8)	
Overweight or obese	164 (20.8)	67 (12.7)	
Physical activity score	10.8 ± 13.9	13.6 ± 15.7	< 0.05
Physical activity level			
Low physical activity	220 (84.6)	411 (77.7)	
Moderate physical activity	27 (10.4)	75 (14.2)	
High physical activity	13 (5.0)	43 (8.1)	

There was a significantdifference in negative body talk scores among university students, with females reporting higher scores than males (t=-2.256, *p* = 0.024; Table 2).

**Table 2:** Sex differences in negative body talk

Variables	Overall (M ± SD)	Men (M ± SD)	Women (M ± SD)	t	*p*
negative body talk score	11.24 ± 6.20	10.53 ± 6.37	11.59 ± 6.09	-2.256	0.024

Body fat was a significant positive predictor of negative body talk (β = 0.26, *P* < 0.001; Table 3). Total happiness score was a significant positive predictor of negative talk (β = 0.09, *P* = 0.002). Restricted diet was a significant positive predictor of negative talk (β = 0.51, *P* < 0.001). Uncontrolled eating was a significant negative predictor of negative talk (β = − 0.08, *P* = 0.013). Emotional eating was not a significant predictor of negative talk (β = 0.06, *P* = 0.083). PARS-3 was not a significant predictor of negative talk (β = 0.04, *P* = 0.152).

**Table 3:** Factors influencing negative body talk among college students

	β	t	VIF	*P*
Body fat	0.26	8.65	1.12	< 0.001
Restricted diet	0.51	15.23	1.43	< 0.001
Total well-being score	0.09	3.17	1.05	0.002
Uncontrolled Eating	-0.08	-2.50	1.43	0.013
Emotional eating	0.06	1.74	1.54	0.083
PARS-3	0.04	1.44	1.06	0.152

R²: 0.413; *P* < 0.001; Root Mean Square Error (RMSE): 22.687

For men: Restricted diet was a significant positive predictor of negative talk (β = 0.49, *P* < 0.001; Table 4). Body fat was a significant positive predictor of negative talk (β = 0.16, *P* = 0.004). Uncontrolled eating was a significant negative predictor of negative talk (β = − 0.14, *P* = 0.036). Total happiness score was not a significant predictor of negative talk (β = 0.10, *P* = 0.093). Emotional eating was not a significant predictor of negative talk (β = 0.03, *P* = 0.652). PARS-3 was not a significant predictor of negative talk (β = 0.03, *P* = 0.527).

**Table 4:** Factors influencing negative body talk among college students of different sexes

	β	t	VIF	*P*
Men≠				
Restricted diet	0.49	6.90	1.68	< 0.001
Body fat	0.16	2.95	1.95	0.004
Uncontrolled Eating	-0.14	-2.11	1.51	0.036
Total happiness score	0.10	1.69	1.11	0.093
Emotional eating	0.03	0.45	1.70	0.652
PARS-3	0.03	0.63	1.02	0.527
Women#				
Body fat	0.36	11.16	1.09	< 0.001
Restricted diet	0.50	13.79	1.36	< 0.001
Total happiness score	0.10	2.93	1.02	0.004
Emotional eating	0.08	2.11	1.47	0.035
Additional diet	-0.02	-0.42	1.34	0.679
PARS-3	-0.01	-0.37	1.01	0.715

≠R²: 0.291; *P* < 0.001; Root Mean Square Error (RMSE): 29.011

#R²: 0.516; *P* < 0.001; Root Mean Square Error (RMSE): 18.013

For women: Body fat was a significant positive predictor of negative talk (β = 0.36, *P* < 0.001). Restricted diet was a significant positive predictor of negative talk (β = 0.50, *P* < 0.001). Total happiness score was a significant positive predictor of negative talk (β = 0.10, *P* = 0.004). Uncontrolled eating was not a significant predictor of negative talk (β = -0.02, *P* = 0.679). Emotional eating was a significant positive predictor of negative talk (β = 0.08, *P* = 0.035). PARS-3 was not a significant predictor of negative talk (β = -0.01, *P* = 0.715).

The results show the characteristics of the three potential subgroups classified by the (LCA) for the university student population and the model fit results (Table 5). After analysing variables such as physical characteristics, dietary behaviours, mental health, and physical activity, the sample was classified into the following three categories:

- Category 1 (high body fat-high restrictive diet group, *n* = 210): this group had the highest percentage of body fat (28.6% ± 6.2) and a significantly higher restrictive diet score (4.2 ± 0.8) than the other groups, but had the lowest total well-being score (12.4 ± 3.2) and level of physical activity (8.3 ± 4.1).
- Category 2 (low body fat-high well-being group, *n* = 350): showed the lowest percentage of body fat (18.4% ± 4.3), the highest well-being (22.1 ± 4.5) and level of physical activity (18.6 ± 5.2), and dietary behavior scores (restrictive diets: 2.1 ± 0.6) were also in the healthy range.
- Category 3 (moderate body fat-moderate diet group, *n* = 229): all indicators fell between the first two categories, such as percentage body fat (23.5% ± 5.1), restricted diet score (3.0 ± 0.7) and physical activity level (12.4 ± 4.7).

**Table 5:** Characteristics of potential category groupings

Variables	Category 1 (high body fat-high restrictive diet group) *n* = 210	Category 2 (low body fat-high well-being group) *n* = 350	Category 3 (moderate body fat-moderate diet group) *n* = 229	*P*
Physical characteristics				
BMI (kg/m²)	25.8 ± 3.5	20.3 ± 2.1	22.7 ± 2.8	< 0.001
Percentage of body fat (%)	28.6 ± 6.2	18.4 ± 4.3	23.5 ± 5.1	< 0.001
Dietary behaviour				
Restricted diet	4.2 ± 0.8	2.1 ± 0.6	3.0 ± 0.7	< 0.001
Emotional eating	3.5 ± 0.9	1.8 ± 0.5	2.7 ± 0.6	< 0.001
Uncontrolled Eating	2.0 ± 0.7	3.5 ± 0.9	2.8 ± 0.8	< 0.001
Mental health				
Total well-being score	12.4 ± 3.2	22.1 ± 4.5	17.6 ± 3.8	< 0.001
Physical activity				
Physical activity score	8.3 ± 4.1	18.6 ± 5.2	12,4 ± 4.7	< 0.001

The results showed that a one-way analysis of variance (ANOVA) revealed significant differences between the three groups on the continuous variables of body fat, eating behaviors and well-being (F = 89.3-142.1, *P* < 0.001), with Bonferroni post hoc tests confirming a significant two-by-two difference between the groups (*P* < 0.05; Table 6). The chi-square test further showed that the ratio of proportion with higher BMI was significantly higher in the high body fat-high restrictive diet group (X² = 156.3, *P* < 0.001).

**Table 6:** Results of the test for differences between groups in potential categories

Variables	Category 1 vs. Category 2	Category 1 vs. Category 3	Category 2 vs. Category 3	F/X²	*P*
Percentage body fat (%)	10.2(28.6 vs. 18.4)	5.1(28.6 vs. 23.5)	-5.1(18.4 vs. 23.5)	268.0	< 0.001
Restrictive diet score	2.1(4.2 vs. 2.1)	1.2(4.2 vs. 3.0)	-0.9(2.1 vs. 3.0)	98.7	< 0.001
Total happiness score	-9.7(12.4 vs. 22.1)	-5.2(12.4 vs. 17.6)	4.5(22.1 vs. 17.6)	121.5	< 0.001
Total physical activity score	-10.3(8.3 vs. 18.6)	-4.1(8.3 vs. 12.4)	6.2(18.6 vs. 12.4)	89.3	< 0.001
Proportion with higher BMI				X²=156.3	< 0.001

The results showed (Table 7) that the negative body talk scores for the high body fat-high dietary restriction group (18.6 ± 4.2) were significantly higher than those for the low body fat-high well-being group (8.3 ± 3.1) and the moderate body fat-moderate dietary restriction group (12.5 ± 3.8), with statistically significant differences (F = 156.3, *P* < 0.001,Table 7).

**Table 7:** Differences in negative body talk scores across potential categories

Variable	Category 1 (High body fat - High dietary restraint group) *n* = 210	Category 2 (Low body fat - High well-being group) *n* = 350	Category 3 (Moderate body fat - Moderate dietary restriction group) *n* = 229	F	*P*
Negative body talk scores	18.6 ± 4.2	8.3 ± 3.1	12.5 ± 3.8	156.3	< 0.001

## Discussion

The present study showed that the participation rate in negative body talk in Southern Chinese college students (68%) was similar to that reported amongEuropean and American adolescents(76%) [2]. However,, there were significant cultural differences in its manifestations and driving mechanisms. In contrast to direct body derogation in Western studies [27], Chinese college students prefer ‘self-deprecating talk’ as a mean to relieve social pressure [28]. Within East Asian collectivist cultures, individuals frequently engage in self-deprecating behaviour to preserve group harmony. Whilst such conduct aims to foster interpersonal cohesion, it inadvertently reinforces societal norms promoting an “ideal of thinness”, thereby exacerbating specific anxieties surrounding body image [29].

Body fat was a significant positive predictor of negative body talk (β = 0.26, P < 0.001), and there was a significant gender difference in negative body talk scores (P = 0.024), with Females scored significantly better compared to males on both body fat percentage and negative body talk. This result reveals the role of the internalisation of social aesthetic standards in shaping sex differences particularly for women. Women have long faced pressure to conform to the ‘thin ideal,’ a challenge that is amplified by social media objectification through labels such as ‘A4 waist’ that heighten sensitivity to body fat [27]. Women’s sensitivity to body fat and dissatisfaction with their body shape making them more likely to engage in negative body talk. In contrast, the weaker negative impact of body fat in men may be related to society’s greater focus on male muscle mass than low body fat [14]. This finding is consistent with global sociocultural pressures for a ‘thin ideal’ for women [30].

The strong positive correlation between restrictive diets and negative body talk (β = 0.51, *P* < 0.001) supports the ‘diet paradox’ theory [10]. Strict calorie control may lead to the depletion of cognitive resources, reducing an individual’s mental toughness in coping with negative evaluations, exacerbating negative talk through self-criticism. Further, although the strength of the effects of restrictive diets was similar for men and women (β = 0.49 vs. 0.50), the underlined motivations seem to differ: men were more motivated by a fitness culture of ‘building muscle and losing fat’ [31], whereas women tended to pursue a ‘slimmer’ diet that conformed to societal expectations. It is advisable to develop gender-specific differentiated intervention strategies—such as providing scientific fitness guidance for men and body acceptance training for women—to alleviate the psychological burden arising from excessive dietary control.

The mitigating effect of uncontrolled eating on negative body talk (β=-0.08, *P* = 0.013) was more pronounced in men (β=-0.14, *P* = 0.036), which may reflect a greater social tolerance for ‘occasional indulgence’ in men. Simultaneously, women faced a high social expectation of ‘self-discipline’ (β=-0.14, *P* = 0.036) [32]. The positive correlation between emotional eating and negative body talk in women (β = 0.08, *P* = 0.035) reveals gender differences in emotion regulation. Women are more likely to eat to alleviate negative emotions, while social stereotypes of women’s emotional expressions may reinforce this behavioural pattern [17]. Emotional eating, while providing short-term relief from psychological stress, may trigger long-term body dissatisfaction because of weight fluctuations, creating a vicious cycle of ‘mood swings-eating-self-blame-negative talk’ [10]. This finding is consistent with Loth KA, who found that women’s emotional eating scores were significantly higher than those of men and were highly correlated with body dissatisfaction [33].

In this study, happiness was found to be significantly positively associated with negative body talk (β = 0.09, *P* = 0.002), indicating hat individuals with a higher level of happiness engaged in negative body talk more frequently. This findingcontradicts the existing literature (or evidence) that happiness alleviates psychological stress in most of the literature [19, 34, 35]. This discrepancy may be due to the specificity of the college student population and the interaction between gender roles and the cultural context. University students find themselves in a challenging transitional phase, grappling with multiple pressures including academic demands, the influence of social media, and peer comparisons regarding body shape [2]. In this context, weight-related discourse may transcend mere venting of personal anxieties, functioning instead as a social strategy: by engaging in self-deprecating fat talk, individuals can gain peer acceptance and integrate into social circles [2, 7]. Consequently, even those with higher levels of well-being may participate in such discussions to conform to group norms and maintain interpersonal harmony.

Sex differences further highlight the implicit pressure of social expectations. The stronger association between well-being and negative talk among women (β = 0.10, *P* = 0.004) reflects the need to balance ‘positive image’ with ‘physical modesty’ [27]. For example, in women who strive to present themselves as both happy and modest—expressed in sentiments such as ‘I am happy, but still need to lose weight’—to avoid being labelled as ‘complacent’ [2, 7, 8]. In contrast, men are more often labelled as ‘complacent’ owing to societal preferences for ‘muscle mass’ [14], and their discussions about weight tend to be less influenced by their subjective well-being.

Furthermore, in East Asian cultures overly positive emotional expressions may be perceived as inconsistent with social norms [36]. Thus, individuals with high level of happiness may strategically balance their emotional expression by using negative language to better conform to cultural expectations. This contrasts with findings from Western research, which identified a negative correlation between well-being and negative body talk [11]. Limitations of measurement tools (e.g., body-specific well-being is not covered by the WHO-5) may mask the cognitive cleavage of ‘holistic well-being-body dissatisfaction’ [19]. In contrast, the Functional Body Appreciation Scale—which measures satisfaction with bodily function rather than appearance—demonstrated a positive association with well-being [37]. This suggests that future research should employ cross-cultural tracking, refine measurement dimensions (incorporating satisfaction with bodily function), and adopt dynamic study designs to elucidate the complex mechanisms linking well-being and weight discussions. Such approaches would provide precise evidence for mental health interventions in higher education settings.

Although the direct impact of physical activity levels on well-being is not significant (β = 0.04, *P* = 0.152), latent category analyses revealed between-group differences, with higher well-being observed in the high-exercise group, suggesting a possible indirect effect. For example, group exercise may improve body image through enhanced social connectedness [37, 38], whereas individual exercise may alleviate anxiety through endorphin release [39].

However, the present study used the PARS-3 scale to quantify exercise intensity without distinguishing between exercise type and social attributes, which may be an essential reason for the non-significant results. Future research is e needed to refine exercise dimensions further to reveal the potential mechanism of physical activity on well-being.

Latent category analysis (LCA) divided the college student population into three subgroups with significant differences: high body fat-high restrictive diet group, low body fat-high well-being group, and moderate body fat-moderate diet group. These clasifications revealed the heterogeneity of college students regarding physical characteristics, eating behaviours, and mental health and further elucidated the differences in the sensitivity of different subgroups to negative body talk.

The high body fat-high restrictive diet group was characterised by a higher percentage of body fat (28.6% ± 6.2) and restrictive diet scores (4.2 ± 0.8) but significantly lower total well-being scores (12.4 ± 3.2) and physical activity levels (8.3 ± 4.1) than the other groups. This group scored the highest on negative body talk, suggesting that the superimposition of high body fat and restrictive dietary behaviours may exacerbate individuals’ anxiety about their body shape, thereby inducing more negative discussions. This is consistent with previous research showing that restrictive diets are often accompanied by excessive focus on weight control, which may trigger social stress and psychological distress [7]. Additionally, lower well-being may weaken an individual’s psychological resilience and make them more vulnerable to negative external evaluations [40]. Comprehensive interventions need to be provided for this group.

The low body fat-high well-being group performed best in terms of percentage body fat (18.4% ± 4.3) and well-being (22.1 ± 4.5), and had significantly higher levels of physical activity (18.6 ± 5.2). This group had the lowest negative body talk scores, suggesting that good physical and mental health may form a protective barrier that reduces sensitivity to weight-related negative evaluations. This finding is consistent with the facilitating effect of well-being on mental health [19], as individuals with high well-being usually possess stronger emotional regulation and can effectively buffer external stress. Such groups can serve as models of healthy lifestyles, and their experiences (e.g., regular exercise and a balanced diet) can inform campus health promotion programs.

Finally, the characteristics of the moderate body fat, moderate diet group, were between those of the previous two groups, and their negative body talk scores were also moderate. Although this group exhibited intermediate levels across indicators such as body fat percentage, eating behaviour and well-being, without displaying pronounced extremes, it nevertheless harbours a latent potential for transitioning towards an unhealthy lifestyle [41]. Moderate health education (information and behavioural guidance delivered at an appropriate intensity and in an accessible manner) alongside social support may assist them in maintaining their health status and preventing future risks (preventing a shift towards increased unhealthy eating behaviours or heightened body dissatisfaction).

## Limitations

There are three limitations to this study. First, the cross-sectional design makes it difficult to infer causality; for example, happiness and negative talk may be causally related. Second, the sample was restricted to students from a single university in Jiangxi Province, which may limit the generalizability of the findings to college populations from other regions or with different cultural backgrounds. Third, it did not incorporate emergent variables, such as social media use, which is becoming necessary for body size comparisons [42]. Future tracking studies could be conducted to capture the dynamics of weight talk in conjunction with the Ecological Method of Instantaneous Assessment (EMA) and to compare differences between groups of different majors and cultural backgrounds.

## Conclusions

Happiness and eating behaviours are essential in reducing negative body talk and negative emotions caused by weight gain among college students. By increasing their well-being and improving eating behaviours, college students can cope better with weight-related stress and negative emotions, improving their mental health and life satisfaction.

## Data Availability

The datasets used and analyzed during the current study are available from the corresponding author on reasonable request.

## Abbreviations

BMI: Body mass index
TFEQ-R21v2: (Twenty-Item Food Intake and Eating Behavior Questionnaire-Revised 21 version 2)
PARS-3: Physical Activity Rating Scale 3
RMSEA: Root mean square of the error of approximation
LCA: Latent Class Analysis
MLR: Monotone Likelihood Ratio family
AIC: Akaike Information Criterion
BIC: Bayesian Information Criterion
SABIC: Sample-Size Adjusted Bayesian Information Criterion
LMRT: Lo-Mendell-Rubin Adjusted Likelihood Ratio Test
BLRT: Bootstrap Likelihood Ratio Test
BCH: Bolck-Croon-Hagenaars
